# Allogeneic Stem Cell Transplantation for Relapsed and Refractory Hodgkin Lymphoma: Real World Experience of a Single Center

**DOI:** 10.3389/pore.2021.1609867

**Published:** 2021-07-27

**Authors:** A. Kopińska, A. Koclęga, A. Wieczorkiewicz-Kabut, K. Woźniczka, D. Kata, M. Włodarczyk, G. Helbig

**Affiliations:** Department of Hematology and Bone Marrow Transplantation, Medical School of Silesia, Silesian Medical University, Katowice, Poland

**Keywords:** Hodgkin lymphoma, allogeneic stem cell transplantation, autologous stem cell transplantation, chemosensitivity, outcome

## Abstract

**Introduction:** Refractory and relapsed Hodgkin lymphoma (R/R HL) is associated with poor prognosis, and allogeneic stem cell transplantation (allo-SCT) remains the only potentially curative approach.

**Aim:** The aim of the study was to evaluate the feasibility of allotransplantation in R/R HL setting.

**Material:** Overall, 24 patients (17 men and 7 women) at a median age of 27 years (range 18–44) underwent allo-SCT between 2002 and 2020.

**Results:** Nineteen patients received prior autologous stem cell transplantation (ASCT1) whereas eight patients received second ASCT (ASCT2) after failure of ASCT1. Six patients received only brentuximab vedotin (BV; *n* = 4) or BV followed by checkpoint inhibitors (CPI; *n* = 2) before entering allo-SCT. Median time from ASCT1 to allo-SCT was 17.1 months. Fifteen patients received grafts from unrelated donors. Peripheral blood was a source of stem cells for 16 patients. Reduced-intensity conditioning was used for all patients. Disease status at transplant entry was as follows: complete remission (CR; *n* = 4), partial response (PR; *n* = 10), and stable disease (SD; *n* = 10). Acute and chronic graft-versus-host disease (GVHD) developed in 13 (54%) and 4 (16%) patients, respectively. Median follow-up for the entire cohort was 13.3 months. At the last follow-up, 17 (71%) patients died. The main causes of death were disease progression (*n* = 10), infectious complications (*n* = 6), and steroid-resistant GVHD (*n* = 1). Non-relapse mortality at 12 months was 25%. At the last follow-up, seven patients were alive; six patients were in CR, and one had PR. The 2-year overall survival (OS) was 40%.

**Conclusion:** Chemosensitive disease at transplant was associated with better outcome. Allo-SCT allows for long-term survival in refractory and relapsed HL.

## Introduction

Standard chemo- and radiotherapy for newly diagnosed Hodgkin lymphoma (HL) results in an ∼80% cure rate, and the remaining patients present primary refractory or relapsed disease (R/R HL). Autologous stem cell transplantation (ASCT) remains a standard therapeutic approach for those with chemosensitive relapse and can be considered as a clinical option for refractory cases [1, 2]. Disease progression or relapse after ASCT is associated with poor prognosis with a median survival of ∼25 months [2, 3]. The broad access to targeted therapies such as an anti-CD30 monoclonal antibody (brentuximab vedotin [BV]) or checkpoint inhibitors (CPI) may overcome disease refractoriness and thereby increase the number of patients who can proceed to allogeneic stem cell transplantation (allo-SCT). The latter is currently recommended for patients who underwent chemosensitive relapse after ASCT but can also be attempted for those with refractory disease after prior unsuccessful targeted treatments [3, 4].

The outcome of allo-SCT seems to be, at least, partly influenced by the type of preparative regimen. The use of myeloablative conditioning (MAC) increases the risk of severe complications and produces a high rate of non-relapse mortality (∼50%). The above mentioned limitations of MAC led to the broader implementation of reduced-intensity conditioning (RIC) which currently remains a standard for allotransplantation in patients with HL [5, 6].

Herein we report on our single center experience with allotransplantation for R/R HL.

## Material and Methods

All eligible patients were identified using our medical records. Twenty-four patients (17 men, 7 women) at a median age of 27 years (range 18–44) underwent allo-SCT between 2002 and 2020. Median age was comparable between genders (*p* = 0.67). Nodular sclerosis was the most common histologic subtype (80%). The Ann Arbor staging system was used for lymphoma staging assessment [7]. Diagnosis was based on histologic examination of the excised lymph node. The following tests were performed in all studied patients: blood film and biochemistry as well as imaging studies including computed tomography (CT) of the whole body and/or positron emission tomography (PET). Trephine biopsy was done when bone marrow infiltration was suspected. Patients were eligible for allo-SCT if they met at least one of the following criteria: 1) primary refractory disease after at least three lines of chemotherapy, 2) early relapse/progression (<12 months) after achieving at least partial response to prior chemotherapy, 3) multiple relapsed patients, and 4) failure of prior ASCT. All patients signed informed consent and the study was conducted in accordance with the Declaration of Helsinki. Ethical review and approval was not required for the study on human participants in accordance with the local legislation and institutional requirements. Allogeneic stem cell transplantation for refractory and relapsed Hodgkin lymphoma remains a standard procedure according to European Society for Blood and Marrow Transplantation (EBMT) recommendations. Characteristics of study patients at diagnosis are shown in Table 1.

**TABLE 1:** Patients’ characteristics.

Parameter	HL (*n* = 24)
Male/female; no	17/7
Median age at diagnosis; (years, range)	24 (15–41)
Histology subtype at diagnosis; no, %	
Nodular sclerosis	19 (80)
Mixed cellularity	5 (20)
Ann Arbor stage; no, %	
II	9 (38)
III	8 (33)
IV	7 (29)
B symptoms; no, %	14 (58)
First-line chemotherapy; *n*, %	
ABVD	15 (63)
MOPP	4 (17)
BEACOPP	4 (17)
ESHAP	1 (3)
Median number of treatment lines; *n*, range	5 (3–10)
Radiotherapy prior to transplantation; *n*, %	20 (83)
ASCT1; *n*, %	20 (83)
ASCT2; *n*,%	8 (33)
Median time from diagnosis to ASCT1; (months, range)	18.3 (9.5–71.1)
Median time from ASCT1 to ASCT2; (months, range)	17.6 (1.7–34.6)*
Median time from diagnosis to allo-SCT; (years, range)	3.97 (1.31–10.8)
Median time from ASCT1 to allo-SCT; (months, range;)	17.1 (3.6–68.0) #

allo-SCT, allogeneic stem cell transplantation; ASCT, autologous stem cell transplantation; HL, Hodgkin lymphoma; *n = 8; #*n* = 20.

### Treatment Prior to Allogeneic Transplantation

First-line chemotherapy consisted of ABVD (adriamycin, bleomycin, vinblastine, dacarbazine; *n* = 15), MOPP (mechlorethamine, vincristine, procarbazine, prednisone; *n* = 4), escalated BEACOPP (bleomycin, etoposide, adriamycin, cyclophosphamide, vincristine, procarbazine, prednisone; *n* = 4), and ESHAP (cisplatin, etoposide, cytarabine, methylprednisolone; *n* = 1). Subsequent salvage lines included different combined regimens. Twenty patients received adjuvant involved field radiotherapy. Twenty patients underwent their first ASCT (ASCT1) after a median of 18.3 months from diagnosis (range 9.5–71.1). The median number of treatment lines before ASCT1 was 4 (range 2–6). Disease status at ASCT1 was as follows: 4 patients achieved second or higher complete remission (CR > 1), 10 were transplanted in partial response (PR) whereas six remained in stable disease (SD). The conditioning consisted of BEAM (carmustine, cytarabine, etoposide, melphalan; *n* = 13), CBV (cyclophosphamide, carmustine, etoposide; *n* = 3), and 4 patients received other regimens. Eight patients received second ASCT (ASCT2) after failure of ASCT1. Median time between ASCT1 and ASCT2 was 17.6 months (range 1.7–34.6). Six patients received only BV (*n* = 4) or BV followed by CPI (*n* = 2) before entering allo-SCT. Among BV-treated patients, the responses were as follows: CR (*n* = 1), PR (*n* = 2), and SD (*n* = 1). Two patients who received CPI achieved PR.

### Response Criteria

The well-recognized response criteria were implemented for response assessment [8].

### Statistical Methods

The probability of overall survival (OS) was assessed using the Kaplan-Meier method. Nonparametric comparisons of group means were performed by using the Mann-Whitney *U* test. Proportions were compared by Fisher exact test. The variables were compared by log-rank test. P value < 0.05 was recognized as statistically significant. The Cox regression model was implemented to evaluate the impact of studied factors on OS. Death before lymphoma progression or recurrence defined non-relapse mortality (NRM). All assessments were done from the date of allotransplantation. The StatSoft software version 12.0 was used for all calculations.

## Results

### Transplant Data

#### Patients Characteristics

Median time from diagnosis and from ASCT1 to allo-SCT was 3.97 years (range 1.3–10.8) and 17.1 months (range 3.6–68.0), respectively. Nine patients received grafts from identical siblings (MRD) and fifteen were transplanted from unrelated donors (MUD). Sixteen patients were transplanted from peripheral blood and eight received stem cells from bone marrow. All transplanted patients were given RIC, usually fludarabine-based. Graft versus host disease (GVHD) prophylaxis consisted of cyclosporine, methotrexate, and anti-thymocyte globulin (the latter was administered for unrelated donors only). The patients exhibited the following lymphoma status at transplant: CR (*n* = 4), PR (*n* = 10), and SD (*n* = 10).

### Posttransplant Outcome

One patient developed primary graft failure (PGF), then underwent second allo-SCT but eventually died due to infectious complications (urosepsis) shortly after the second allotransplantation. All other patients were engrafted after a median of 18 days (range 13–24). Platelet count of >20 × 10^9^/L was demonstrated for 21 patients after median of 13 days (range 8–23). Median time between the date of allo-SCT and onset of acute GVHD was 18 days (range 10–44). A total of 13 (54%) and 4 (16%) transplanted patients developed acute and chronic GVHD, respectively. Four subjects demonstrated acute GVHD grade II-IV whereas extensive chronic GVHD was observed in two. Details on posttransplant outcome are presented in Table 2.

**TABLE 2:** Transplant details.

Parameter	HL (*n* = 24)
Median age of recipient at transplant; years, range	27 (18–44)
Disease status at allo-SCT; *n*, %	
CR	4 (16)
PR	10 (42)
SD	10 (42)
Median age of donor at transplant; years, range	30 (19–51)
Type of donor; *n*, %	
Matched sibling donor	9 (38)
Unrelated donor	15 (62)
Stem cell source; *n*, %	
Bone marrow	7 (29)
Peripheral blood	16 (67)
Bone marrow and peripheral blood	1 (4)
Donor-recipient sex matching; *n*, %	
Donor male-recipient female	4 (16)
Donor female-recipient male	6 (25)
Sex matching	14 (58)
ABO-blood group matching; *n*, %	
Matched	10 (42)
Minor mismatch	9 (38)
Major mismatch	3 (13)
Minor and major mismatch	2 (7)
Type of conditioning; no, %	
BuFlu	16 (67)
FluMelAlem	5 (21)
MelTBI	2 (8)
BEAM	1 (4)
GVHD prophylaxis; no, %	
CsA + Mtx	19 (79)
MMF + Mtx	3 (13)
TAC + MMF	1 (4)
TAC + MMF+ post Cy	1 (4)
Median number of transplanted CD34-positive cells (x10^6^/kg); range	5.0 (1.37–9.32)
Median number of transplanted CD3-positive cells (x107/kg); range	14.4 (1.46–52.4)
Median ANC>0.5 (x10^9^/L); days, range	18 (13–24)
Median PLT >20 (x10^9^/L); days, range	13 (8–23)
Disease status of survivors; *n*, %	7 (100)
CR	6 (86)
PR	1 (14)

allo-SCT, allogeneic stem cell transplantation; ANC, absolute neutrophil count; BEAM, carmustine, etoposide, cytarabine, melphalan; BuFlu, busulphan, fludarabine; CR, complete remission; CsA, cyclosporine; Cy, cyclophosphamide; FluMelAlem, fludarabine, melphalan, alemtuzumab; GVHD, graft versus host disease; MelTBI, melphalan, total body irradiation; MMF, mycofenolan mofetil; Mtx, methotrexate; PLT, platelets; PR, partial response; SD, stable disease; TAC, tacrolimus.

The following infectious complications were demonstrated in the early post-transplant period: bacterial pneumonia (*n* = 2), pulmonary aspergillosis (*n* = 1), urosepsis (*n* = 1), and BKV cystitis with hematuria (*n* = 2). Four deaths were noted up to day +100 after allo-SCT and they were as follows: pulmonary aspergillosis (*n* = 1), bacterial pneumonia (*n* = 2), and disease progression (*n* = 1).

**FIGURE 1:**
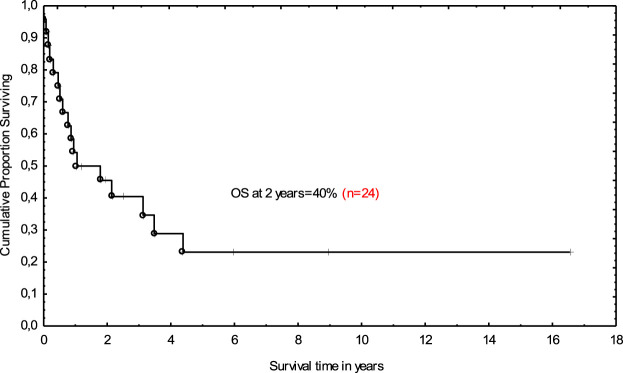
Overall survival for allotransplanted patients with Hodgkin lymphoma.

**FIGURE 2:**
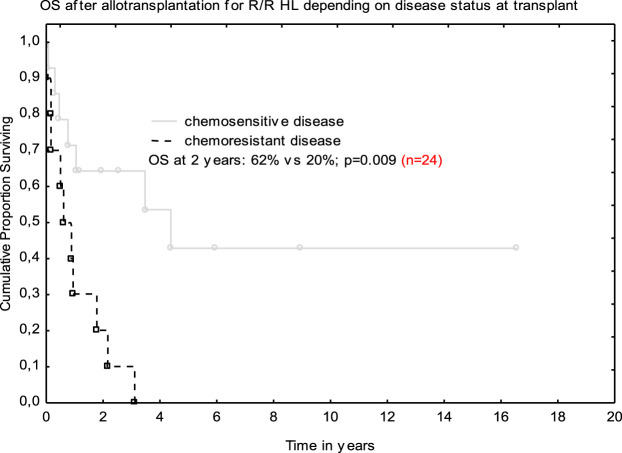
Overall survival after allo-SCT depending on disease status at transplant.

Post-allograft disease assessment on day +100 was performed in 20 patients and CR was achieved in 8 patients, PR in 2, and 10 patients demonstrated disease progression or stabilization. Median follow-up for the entire cohort was 13.3 months (range 0.1–195). In total, 17 (71%) patients died. The main causes of death were lymphoma progression (*n* = 10), severe infections (*n* = 6), and resistant GVHD (*n* = 1). Non-relapse mortality at 12 months was 25%. At the last follow-up, seven patients were alive; CR was maintained in six patients, and one individual remained in PR. All those patients had full donor chimerism. The 2-year OS was 40% (Figure 1). Median follow-up for survivors was 5.9 years (range 1.1–16.5). Tendency for better outcome was demonstrated for male patients who received ASCT1 and developed acute GVHD. Only patients with chemosensitive disease at transplant had better survival when compared with those with stable disease in multivariate analysis; 62 vs. 20% at 2 years; (HR 0.47 [95% CI; 0.27–0.82]; *p* = 0.009 (Figure 2).

## Discussion

Relapsed and refractory HL is undeniably associated with poor prognosis, and allo-SCT remains the only potentially therapeutic option. Of note, the introduction of BV/CPI induced a deeper clinical response in those who failed prior ASCT and increased the number of patients who could proceed to allotransplantation [9, 10]. It was demonstrated that BV/CPI used as a “bridge” to transplantation may offer survival benefits in this poor prognosis population. Patients who achieved CR after BV had an estimated 5-year OS of 64% and the median OS was not reached. A proportion of these patients proceeded to allo-SCT and remained in sustained CR [11]. In turn, OS was higher in patients who responded to CPI and received allotransplantation [10]. It should be highlighted that BV/CPI have only been available for Polish HL patients in recent years. In our series, only six patients were treated with BV/CPI before transplant; 5/6 patients demonstrated chemosensitivity to BV/CPI whereas 9/18 individuals responded to conventional chemotherapy (OS was comparable between these subgroups with *p* = 0.12, data not shown). Regarding the sustained long-term response to these newer agents, the question of to whom and when to offer an allotransplantation remains open. Safety aspects in patients undergoing allotransplantation proceeded by administration of CPI are of a special concern. Namely, it was demonstrated that pre-transplant use of CPI may increase the incidence of GVHD and the risk of other immune-mediated complications [12]. Interestingly, the administration of posttransplant cyclophosphamide may alleviate the incidence and severity of GVHD [13]. Of note is that one of our CPI-treated patients developed fatal grade IV acute GVHD which remained resistant to all available immunosuppressive agents despite posttransplant cyclophosphamide. Given the high efficacy of novel agents, the question of whether we should postpone allotransplantation remains open. In the Chen study [11], median OS/PFS were not reached for RR HL patients who achieved CR following BV treatment; OS/PFS were 64 and 52%, respectively. This study has demonstrated that some patients may have a sustained response on BV and even be cured without transplant consolidation. Thus, it was postulated to postpone transplantation until disease progression occurred [11]. In relation to CPI-treated patients, the decision when to proceed to transplantation also remains unclear, especially keeping in mind their immunomodulatory and a partially detrimental effect. One-year incidence of grades II-IV acute GVHD and chronic GVHD was 44 and 41%, respectively. Moreover, some patients developed fatal sinusoidal obstruction syndrome [12]. In the context of current knowledge, it is reasonable to use CPI as a salvage treatment for those who failed BV, however one should bear in mind that their use before transplantation may be associated with increased toxicity. Recently, a large retrospective dataset on the outcome of 209 transplantations proceeded by CPI treatment has been published. After a follow-up of 2 years, GVHD, PFS, and OS were 47, 69, and 82%, respectively. NRM was 14%. Multivariate analysis has demonstrated that the incidence of GVHD was reduced in patients with a longer interval between CPI treatment and transplantation [14].

It was proved that the attainment of chemosensitivity before transplantation remains the main goal of our therapeutic approaches and translates into the success of the procedure [9–11, 15]. That was also true for our small analysis. Namely, the patients with chemosensitive disease fared much better than those who remained chemoresistant—OS at 2 years was 62 vs. 20%, respectively. The 2-year OS for the entire group was 40% and it was in line with other studies [15].

Historically, the use of myeloablative regimens (MAC) showed a high rate of NRM with a low probability of 3-year OS [16]. A retrospective EBMT study on 168 HL patients compared the outcomes of RIC vs. MAC and it was found that the latter was associated with significantly higher NRM which translated into lower OS. On the other hand, the patients in the RIC group showed a higher relapse rate when compared to the MAC group [6]. With modern transplant practices, the difference in NRM between RIC and MAC has been vanishing. The large EBMT study with 312 patients transplanted between 2006 and 2010 did not show a significant difference in NRM between RIC and MAC. There was a lower relapse rate and tendency of better event-free survival in MAC, however OS was comparable [17]. Our study patients received mostly fludarabine-based RIC conditioning so it is difficult to draw any conclusions. Of note, five patients from our study were given fludarabine with melphalan and alemtuzumab plus cyclosporine as GVHD prophylaxis. These patients did not display the symptoms of severe GVHD but eventually died of disease progression (*n* = 2) or infectious complications (*n* = 2). Only one patient is still alive. This combination was compared with cyclosporine/methotrexate in a study performed by United Kingdom and Spanish collaborative groups and found to decrease NRM and the incidence of GVHD with no increase of relapse rate. There was also a tendency of a longer duration of response in the alemtuzumab group [18].

Some authors determined the impact of donor types on the posttransplant outcomes in HL patients, but data are somehow conflicting. Two-year OS and PFS were comparable between different donor types, however NRM was lower for haploidentical (HAPLO) recipients when compared with MRD. Moreover, HAPLO transplants had lower relapse incidence (RI) vs. MRD and MUD [19]. Patients who received HAPLO grafts were found to have comparable incidence of acute GVHD, but a lower risk of chronic GVHD than MUD. NRM was comparable between HAPLO and MRD, but lower than in MUD [20]. In a large study of 596 multi-treated HL patients, the outcome of HAPLO versus MRD was compared. No significant difference in terms of OS and PFS was demonstrated between the groups, however those who received grafts from HAPLO donors had a significantly higher risk of grades II-IV acute GVHD (but not grades III-IV), decreased incidence of chronic GVHD, and reduced RI. There was a trend toward higher NRM in the HAPLO cohort [21]. In the Castagna study [22], with 198 HL patients in CR, HAPLO was associated with significant better 2-year PFS when compared with MRD, no difference was found in terms of OS. It was also demonstrated that PFS, OS, and RI were better when the patient was transplanted in CR than in PR. Based on the current experience, HAPLO is feasible in HL patients with efficacy and safety comparable to MRD and MUD.

In our group, we did not show any difference in OS and GVHD incidence between transplants from related and unrelated donors (*p* = 0.73 and 0.67, respectively, data not shown). The source of stem cells (bone marrow vs. peripheral blood) displayed no impact on OS as well as age at transplant. HAPLO was not performed.

It should be mentioned that our analysis carries some limitations related to the retrospective nature of the study: a relatively small number of included patients and a low proportion of BV/CPI-treated patients. Nevertheless, long-term remission can be achieved in a proportion of HL patients especially in those with chemosensitive disease.

In summary, a meta-analysis of outcomes of allo-SCT performed in 1850 RR HL patients demonstrated a better PFS/OS and lower RR and NRM over time with a significant improvement in those transplanted in 2000 and later when compared with earlier studies [23]. The Lymphoma Working Party of the EBMT (European Society for Blood and Marrow Transplantation) has focused on the changes in allotransplantation for RR HL over the last 25 years. The number of allo-SCT has increased over time, however it can be partially explained by the higher number of transplant centers and reporting countries. Nowadays, transplanted patients are older and have better performance status. The time between diagnosis and transplantation has shortened. Most patients are transplanted in chemosenstive disease. Peripheral blood remains a main source of stem cells and RIC is preferred over MAC. Other sources than sibling donors are used for transplantations including HAPLO. The substantial improvement in PFS, OS, and NRM has been documented, but relapse after transplantation still remains a main challenge. The incidence of severe acute GVHD has decreased whereas extensive chronic GVHD remains stable [24]. In relation to our cohort, the number of transplantations was stable during the analysis period (between 2 and 4 transplants yearly). Six patients were transplanted in the recent 5 years (2015–2020) and they were significantly older than those transplanted earlier (35 vs. 26 years; *p* = 0.04, data not published). Peripheral blood prevailed as a source of stem cells for transplantation and bone marrow was last used in 2011. RIC predominates, and alemtuzumab as a part of conditioning was discontinued in 2006. All patients transplanted between 2015 and 2020 were chemosensitive at transplant (at least PR) which translated into a tendency of better OS (*p* = 0.07). Incidence of GVHD was comparable. There was no difference in time between diagnosis and transplantation over time (*p* = 0.72).

## Conclusion

Allogeneic stem cell transplantation allows for long-term survival in refractory and relapsed HL. The attainment of chemosensitivity before transplantation provides better survival.

## Data Availability

The raw data supporting the conclusion of this article will be made available by the authors, without undue reservation.
